# The burden of metabolic syndrome on osteoarthritic joints

**DOI:** 10.1186/s13075-019-2081-x

**Published:** 2019-12-16

**Authors:** Bruce M. Dickson, Anke J. Roelofs, Justin J. Rochford, Heather M. Wilson, Cosimo De Bari

**Affiliations:** 1 Institute of Medical Sciences, University of Aberdeen, Aberdeen, UK; 2 The Rowett Institute, University of Aberdeen, Aberdeen, UK

**Keywords:** Osteoarthritis, Metabolic syndrome, Obesity, Macrophage, Chondrocyte

## Abstract

**Background:**

The prevalence of osteoarthritis (OA) increases with obesity, with up to two thirds of the elderly obese population affected by OA of the knee. The metabolic syndrome (MetS), frequently associated with central obesity and characterised by elevated waist circumference, raised fasting plasma glucose concentration, raised triglycerides, reduced high-density lipoproteins, and/or hypertension, is implicated in the pathogenesis of OA. This narrative review discusses the mechanisms involved in the influence of MetS on OA, with a focus on the effects on macrophages and chondrocytes.

**Main text:**

A skewing of macrophages towards a pro-inflammatory M1 phenotype within synovial and adipose tissues is thought to play a role in OA pathogenesis. The metabolic perturbations typical of MetS are important drivers of pro-inflammatory macrophage polarisation and activity. This is mediated via alterations in the levels and activities of the cellular nutrient sensors 5′ adenosine monophosphate-activated protein kinase (AMPK) and mammalian target of rapamycin complex 1 (mTORC1), intracellular accumulation of metabolic intermediates such as succinate and citrate, and increases in free fatty acids (FFAs) and hyperglycaemia-induced advanced glycation end-products (AGEs) that bind to receptors on the macrophage surface. Altered levels of adipokines, including leptin and adiponectin, further influence macrophage polarisation. The metabolic alterations in MetS also affect the cartilage through direct effects on chondrocytes by stimulating the production of pro-inflammatory and catabolic factors and possibly by suppressing autophagy and promoting cellular senescence.

**Conclusions:**

The influence of MetS on OA pathogenesis involves a wide range of metabolic alterations that directly affect macrophages and chondrocytes. The relative burden of intra-articular versus systemic adipose tissue in the MetS-associated OA remains to be clarified. Understanding how altered metabolism interacts with joints affected by OA is crucial for the development of further strategies for treating this debilitating condition, such as supplementing existing therapies with metformin and utilising ω-3 fatty acid derivatives to restore imbalances in ω-3 and ω-6 fatty acids.

## Introduction

Osteoarthritis (OA) is a painful and debilitating degenerative joint disease characterised by progressive loss of articular cartilage, synovitis, subchondral bone sclerosis, and osteophyte formation. An increasing body of evidence indicates that chronic low-level inflammation plays an important role in the pathogenesis of OA. The presence of synovitis, characterised by infiltration of immune cells, angiogenesis, and synovial hypertrophy/hyperplasia, has been linked not only to increased joint pain but also to disease progression [1, 2].

The prevalence of OA increases with obesity, with up to two thirds of the elderly obese population affected by OA of the knee [3]. The metabolic syndrome (MetS), frequently associated with central obesity, could promote inflammatory processes implicated in the pathogenesis of OA. This narrative review will discuss the influence of MetS and obesity on OA, with a specific focus on the mechanisms through which MetS can influence inflammatory processes, particularly the activation and polarisation of macrophages, including perturbations in cellular nutrient sensing, adipokine production, and long-chain fatty acids. It will also appraise the role of weight loss in the management of OA and the potential of dietary fatty acids as targets for future therapies.

## Methods

A literature review was conducted through an electronic search of MEDLINE and PubMed search engines to identify relevant papers using the following keywords: osteoarthritis, obesity, metabolic syndrome, hyperglycaemia, hypertension, infrapatellar fat pad, macrophage, and chondrocyte. Further electronic searches were conducted for specific proteins of interest that emerged from the initial searches. Additional references were identified from reference lists to supplement electronic searching. Final references for inclusion were selected to provide substantial coverage of the reviewed topics, whilst adhering to the editorial guidelines regarding the number of citations.

## Association between MetS and OA

### Evidence from epidemiological studies

MetS can be defined as the presence of any three of the following risk factors: elevated waist circumference, raised fasting plasma glucose concentration, raised triglycerides, reduced high-density lipoproteins, or hypertension [4]. MetS is accompanied by chronic low-grade systemic inflammation [5]. The clinical importance of this has been increasingly recognised, with conditions driven by chronic inflammation such as psoriasis linked to MetS [6]. For OA, the strongest epidemiological links are found between MetS and OA of the knee [7]. A greater body mass, commonly associated with MetS, and a resulting increase in the forces acting on the load-bearing joints may be in part responsible for this association. However, obesity also increases the risk of development of OA in non-weight-bearing joints of the hand [7]. Irrespective of a patient’s BMI, hyperlipidaemia and hypertension as individual MetS components have been linked to the development of OA [8]. These and other studies [9] thus provide evidence linking MetS to OA independent of a patient’s BMI.

### Evidence from preclinical studies and the role of inflammation

The link between MetS and OA is supported by preclinical studies. A commonly used experimental model in rodents is a high-fat diet (HFD), which leads to obesity, hyperglycaemia and dyslipidaemia, and OA. HFD can also exacerbate post-traumatic OA in mice [10]. Wheel-running exercise, and hence increased biomechanical forces exerted on joints, protected from HFD-induced OA, and this was associated with improved glucose tolerance without reducing body fat [11]. This indicates that biomechanics alone cannot account for the worsening of OA, at least in rodents, and that exercise may be joint protective through improving metabolic function. In addition, HFD-induced OA is associated with systemic elevations in pro-inflammatory cytokines [11]. Local adipose tissue such as the infrapatellar fat pad (IFP) in the knee may also produce inflammatory and catabolic mediators that contribute to OA pathogenesis and has been implicated as a source of inflammatory cytokines in both murine HFD-induced OA [12] and in human rheumatoid arthritis and OA [13]. Indeed, the IFP from OA patients was shown to have significantly increased levels of IL-6, monocyte chemoattractant protein-1 (MCP-1), vascular endothelial growth factor (VEGF), and leptin, whilst also showing increased levels of fibrosis compared to healthy controls [14]. Furthermore, when compared to autologous subcutaneous fat, intra-articular adipose tissue within the infrapatellar and suprapatellar and acetabular fat pads all showed significantly increased levels of vascularity, fibrosis, and inflammatory mediators in OA patient samples [15]. It must be noted that OA changes within the intra-articular adipose tissues were unaffected by the presence of obesity. Similarly, other studies have disputed whether HFD leads to changes within the IFP, with no evidence found for an increase in inflammation nor adipocyte hypertrophy in the IFP in response to HFD, as had been observed in epididymal fat [16]. This suggests that the IFP may not undergo similar inflammatory changes in response to HFD as abdominal adipose tissue. It is increasingly recognised that differences in cellular composition and metabolic function exist between adipose depots. This is potentially a result of adipocyte populations arising from differing embryological sources [17] and the heterogeneous nature of the progenitor cell populations found within individual depots [18]. Intriguingly, these populations undergo significant alterations in the presence of diabetes [18]. Whilst research into the diverse nature of adipocyte biology is ongoing, our knowledge regarding intra-articular adipose tissue is particularly limited in relation to its insulin responsiveness, lipid handling properties, and response to inflammation. Thus, the relative importance of local versus systemic adipose inflammation, and of metabolic dysregulation, in the MetS-associated OA remains to be clarified.

## Macrophages as key effector cells in OA

Infiltrating macrophages are key cells in the inflammatory processes. They are heterogeneous cells that exhibit remarkable plasticity, able to adopt many phenotypes and functions dependent on the microenvironmental signals they receive. Knowledge of the range of macrophage activation states and the cues that induce polarisation is far from complete, but two broad types of macrophage activation have been characterised: M1-activated macrophages are induced by pro-inflammatory stimuli, e.g. TNF-α, and have anti-microbial and cytotoxic properties that can damage tissue and rely heavily on glycolysis to meet their energy demands. By contrast, M2-activated macrophages are anti-inflammatory or reparative and use oxidative phosphorylation to provide a sustained ATP energy provision. A spectrum of activation states intermediate to these is found in infiltrating macrophages in vivo [19].

Early evidence for macrophages playing a key role in the development of OA came from murine studies utilising liposomal clodronate to selectively ablate macrophages. Osteophyte formation in a model of collagenase-induced OA was significantly reduced by up to 84% when macrophages were ablated prior to inducing OA [20], which was suggested to be due to the reductions in bone morphogenetic protein (BMP) 2 and 4 production by synovial lining macrophages [20]. In addition, macrophage ablation resulted in reduced levels of MMP2, MMP3, and MMP9 and decreased cartilage breakdown [21]. These experiments indicate the importance of macrophages in the disease process.

Activated macrophages have since been shown to be directly involved in the development of synovitis in human OA as visualised by etarfolatide-enhanced single-photon emission computed tomography-computed tomography (SPECT-CT) [22]. Etarfolatide binds only to the functional form of folate receptor β, expressed abundantly on activated macrophages but not on resting macrophages. OA patients were shown to have increased numbers of activated synovial macrophages compared to healthy controls, and macrophage numbers were significantly associated with pain and joint space narrowing [22]. It was further shown that elevated soluble biomarkers, CD14 and CD163, indicative of macrophage activation, in the synovial fluid were significantly associated with increased abundance of activated synovial membrane macrophages when compared to the etarfolatide scanning results, worsening patient pain scores, and progression of OA as measured by Kellgren-Lawrence plain radiograph severity scoring [23]. Thus, these studies not only indicate that macrophages play an important role in the underlying disease process but that biomarkers of activated macrophages may be able to predict patients at high risk of disease progression.

**Fig. 1:**
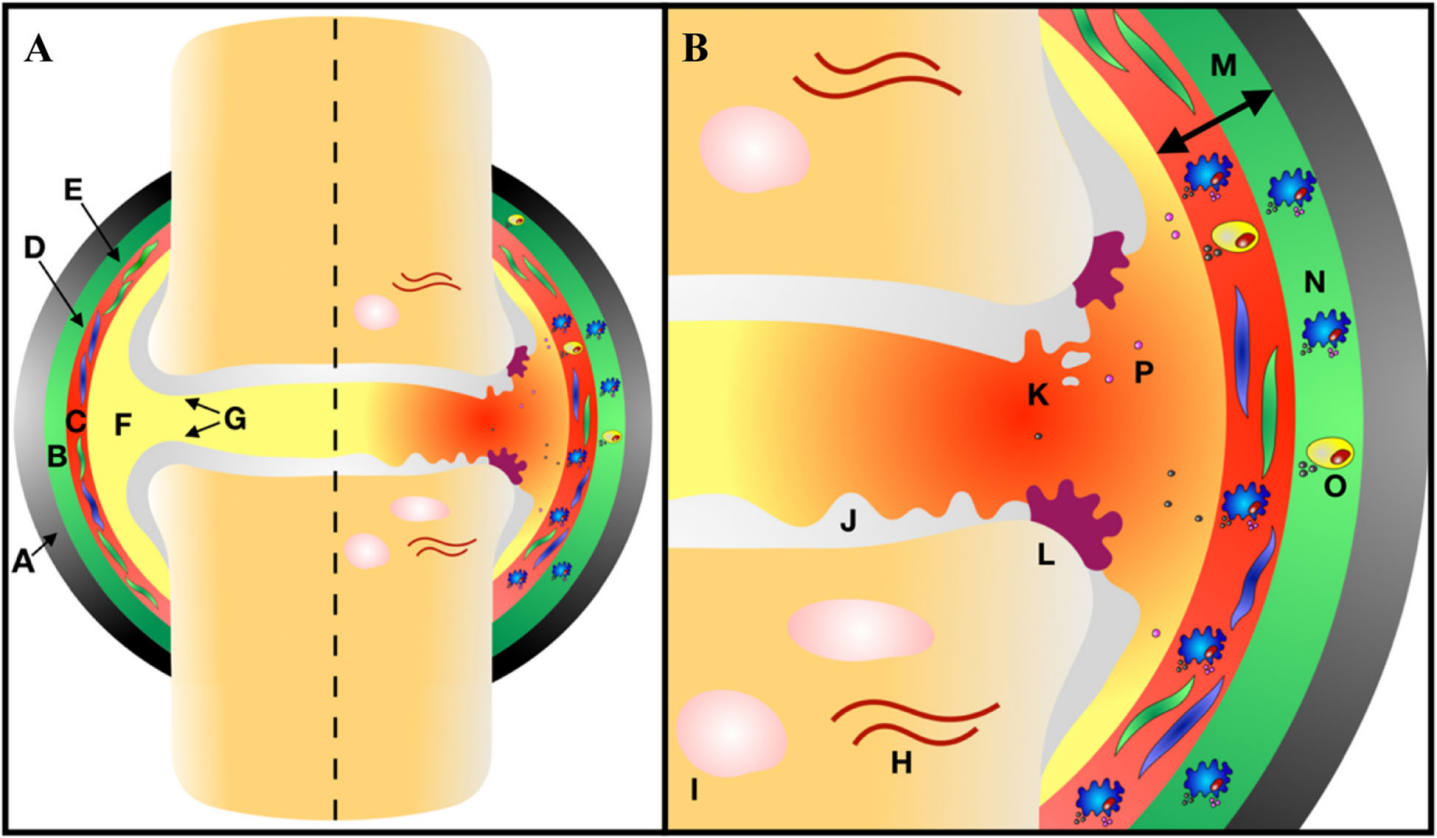
Chronic inflammation in osteoarthritis. **a** (Left) healthy synovial joint. Type A and B synoviocytes present within intimal synovial lining. The cartilage covering the articulating surface of bones. (Right) chronic inflammation within the synovial joint. **b** Expanded view of chronic inflammation. DAMPs released from the cartilage and synovium result in synoviocyte activation. Macrophages and CD4+ T cells infiltrate the synovium resulting in the release of pro-inflammatory mediators and chronic inflammation. The cartilage and bone are degraded and remodelled with subchondral sclerosis and osteophyte and cyst formation. (A) Fibrous capsule, (B) subintimal synovial lining, (C) intimal synovial lining, (D) type A synoviocyte, (E) type B synoviocyte, (F) synovial fluid, (G) cartilage, (H) subchondral sclerosis, (I) subchondral cyst, (J) cartilage degradation, (K) DAMP release, (L) osteophyte formation, (M) synovial hypertrophy, (N) macrophage infiltration, (O) CD4+ T cell infiltration, and (P) pro-inflammatory mediator secretion

It has been proposed that the activation and infiltration of macrophages into the synovium is brought about by an initial insult to the joint, releasing damage-associated molecular patterns (DAMPs) that are recognised by a selection of pattern recognition receptors (PRRs) expressed on macrophages (Fig. 1). Macrophage recognition of DAMPs, including high-mobility group box-1 (HMGB1), S100A8 alarmins, and MMPs, leads to their activation, transcription of NF-κB, and subsequent production of pro-inflammatory mediators such as TNF, interleukin (IL)-1β, and IL-6 [24]. This release of pro-inflammatory mediators leads to the activation of fibroblast-like synoviocytes and production of MMPs and disintegrin and metalloproteinases with thrombospondin motifs (ADAMTS), which cause degradation of the cartilage through cleavage of aggrecan and other cartilage matrix proteins [25]. Meanwhile, both activated macrophages and fibroblasts release chemotactic proteins such as chemokine ligand (CCL) 2, CCL3, and CCL4 inducing infiltration of circulating monocytes and CD4^+^ T cells into the synovium, where the former differentiate into macrophages [26]. As well as DAMPs, metabolic intermediates can polarise macrophages to different functional states that affect their roles in OA. A study characterising the phenotype of macrophages isolated from synovial fluid showed that OA patients have a preponderance to an M1/M2 imbalance with a greater ratio of M1/M2 correlating with increasing severity of radiographic OA [27].

## The effect of MetS on macrophage polarisation

Macrophages are present in metabolic tissues such as fat, liver, and muscle, and their proliferation, plasticity, and polarisation are driven by obesity, with a switch from the M2 to M1 phenotype being observed [28]. Preclinical studies have demonstrated a skewing of macrophages towards the M1 phenotype within synovial and adipose tissues in diet-induced OA [16]. There are several molecular mechanisms through which MetS could promote a pro-inflammatory M1 macrophage phenotype in OA, including metabolic perturbations at the cellular level and changes in the systemic factors such as adipokine levels.

### Metabolic programming of macrophage polarisation

**Fig. 2:**
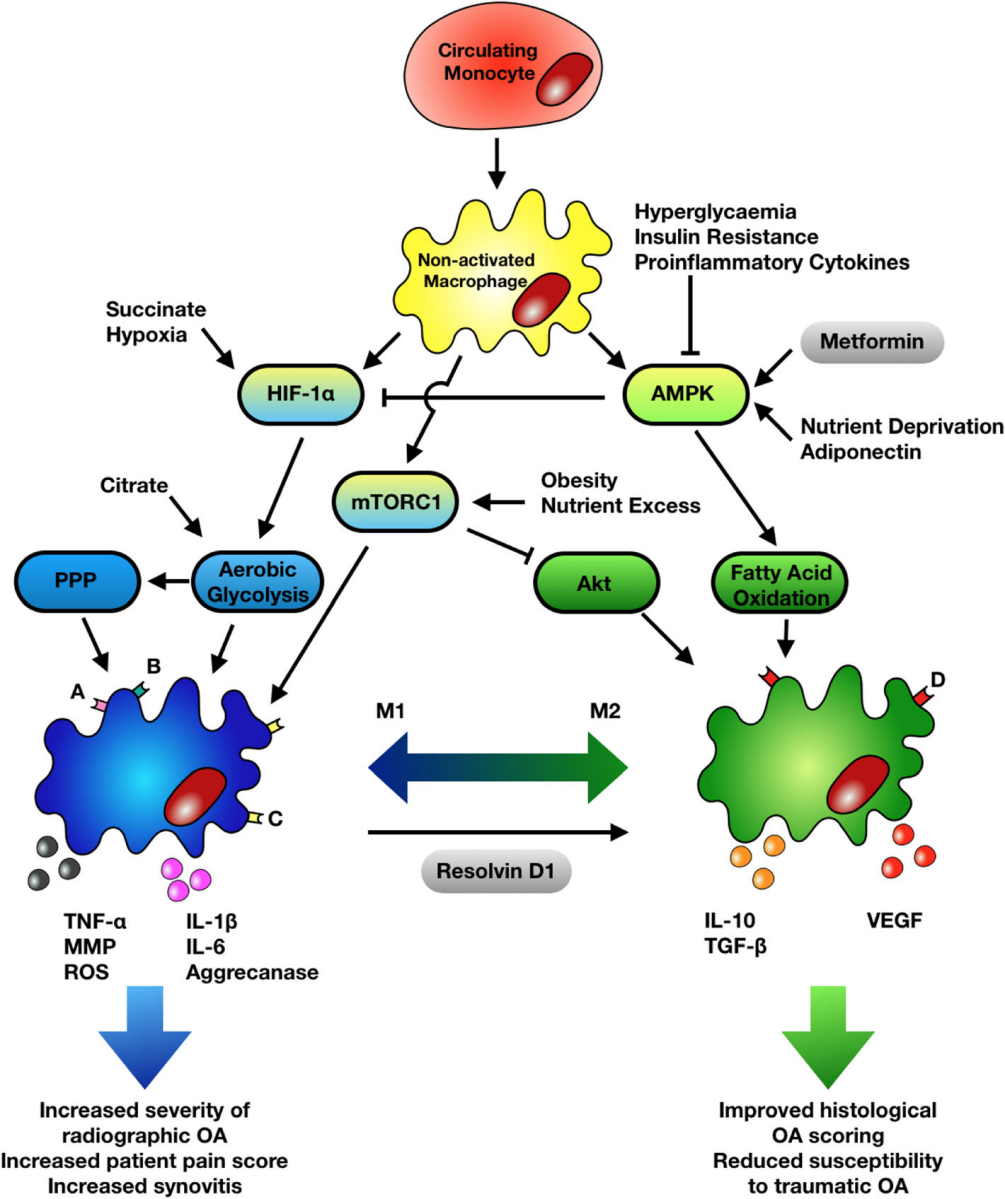
Metabolic polarisation of macrophages. Circulating monocytes are recruited into the synovium whereby they differentiate into non-activated macrophages. Hyperglycaemia, insulin resistance, and pro-inflammatory cytokines inhibit AMPK activity resulting in HIF-1α stabilisation and increases in aerobic glycolysis. Increases in glycolysis are accompanied by increased PPP activity, and both are involved in M1 macrophage polarisation. Succinate stabilises HIF-1α. Citrate promotes aerobic glycolysis and inflammatory cytokine expression. Obesity and nutrient excess hyperactivate mTORC1 resulting in Akt inhibition and defective M2 polarisation. M2 polarisation is promoted by AMPK activity. AMPK is stimulated by nutrient deprivation, metformin, and adiponectin. Resolvin D1 promotes the re-polarisation of macrophages to the M1 phenotype. AMPK, 5′ adenosine monophosphate-activated protein kinase; HIF-1α, hypoxia-inducible factor alpha; PPP, pentose phosphate pathway; mTORC1, mammalian target of rapamycin complex 1; TNF-α, tumour necrosis factor alpha; MMP, matrix metalloproteinase; ROS, reactive oxygen species; IL, interleukin; TGF-β, transforming growth factor beta; VEGF, vascular endothelial growth factor. (A) CD11c, (B) CD14, (C) CD86, and (D) CD206

Metabolic perturbations, including changes in the levels of oxygen, nutrients, and extracellular metabolites, are perceived by immune cells including macrophages through the activity and levels of the nutrient sensors 5′ adenosine monophosphate-activated protein kinase (AMPK), and mammalian target of rapamycin complex 1 (mTORC1). The activity of AMPK plays a key role in metabolic reprogramming in response to nutrient deprivation (Fig. 2), via its ability to sense falling intracellular glucose and ATP levels. AMPK activity subsequently increases ATP production whilst reducing anabolic processes to restore cellular energy homeostasis [29]. AMPK activity is reduced by several aspects of MetS including insulin resistance, hyperglycaemia, and elevated circulating pro-inflammatory mediators. A reduction in AMPK activity in macrophages increases aerobic glycolysis by stabilising hypoxia-inducible factor-1α (HIF-1α) via the Warburg effect. Increased glycolysis in macrophages is associated with a pro-inflammatory phenotype as it produces more glucose-6-phosphate (G6P), the main substrate of the pentose phosphate pathway (PPP), allowing the production of NADPH that is used to generate reactive oxygen species (ROS) [30], implicated in immune cell activation and in the damage of chondrocytes. Indeed, G6P-dehydrogenase (G6PD), the first enzyme within the PPP, has been shown to be upregulated in macrophages derived from obese patients and, along with NADPH, to be essential for the activation of NF-κB and ROS formation [31].

The nutrient sensor mTORC1 integrates signals from multiple sources, including cellular energy levels, oxygen status, growth factors, and amino acid availability, and is responsible for anabolic processes including protein, lipid, and nucleotide synthesis. Obesity and nutrient excess are known to induce the hyperactivation of mTORC1, which leads to defective M2 polarisation of macrophages via feedback inhibition of the serine-threonine kinase Akt [32]. Akt is responsible for upregulating many of the genes essential in M2 polarisation such as *Arg*1, *Fizz*1, and *Ym*1 whilst at the same time promoting the inhibition of M1 polarisation through downregulating transcription factor FOXO1, essential for PRR, Toll-like receptor 4 (TLR4) production and upregulating IL-1 receptor-associated kinase M (IRAK-M), a TLR4 signalling inhibitor [32]. Similar effects were observed in a murine OA model. Myeloid lineage-specific deletion of tuberous sclerosis complex 1 (TSC1) led to hyperactivation of mTORC1 and was associated with M1 polarisation of synovial macrophages with resultant increases in IL-1, IL-6, and TNF [33]. This skewing to the M1 phenotype was accompanied by worsening of OA. Furthermore, in Rheb1 deletion mice where mTORC1 is constitutively inactive in the myeloid lineage, it led to M2 macrophage polarisation within the synovium accompanied by improvements in OA histological severity. A recent study in rheumatoid arthritis further highlights the detrimental effects that altered AMPK and mTORC1 activity can have on synovial inflammation via effects on T cells. T cells from RA patients were shown to have deficient N-myristoylation, a lipid modification of proteins that changes their physical properties and their subcellular distribution [34]. Defective N-myristoylation of AMPK prevented its activation and instead led to exuberant mTORC1 signalling, stimulating differentiation into pro-inflammatory T_H_1 and T_H_17 T cells and promoting inflammation in a humanised mouse model of synovitis [34]. Whether metabolic reprogramming affects T cells in OA remains to be determined.

MetS can also impact on crucial metabolites involved in macrophage polarisation and activity. One of these metabolic intermediates is succinate. It increases not only due to the Krebs cycle stalling in M1 macrophages but also in response to hyperglycaemia and obesity. Succinate has been shown to compete with prolyl hydroxylase resulting in the stabilisation of HIF-1α within macrophages with subsequent sustained production of IL-1β through directly binding to the *Il1b* promoter [35]. The stalled Krebs cycle causes the accumulation of a further intermediate, citrate, within the mitochondria that is crucial to M1 effector function. Citrate is exported out of the mitochondria and is further metabolised to acetyl-CoA, vital in the acetylation of histones regulating not only the transcription of glycolytic enzymes, needed to increase energy production in the M1 macrophage, but also of inflammatory cytokines such as IL-6 [36].

### Macrophage polarisation induced by AGEs and FFAs

In addition to affecting key nutrient sensors and metabolic intermediates that polarise macrophages, the MetS can influence macrophage function via advanced glycation end-products (AGEs) and free fatty acids (FFAs) that act directly on macrophages. Chronic hyperglycaemia non-enzymatically glycates proteins and lipids and thus produces advanced glycation end-products (AGEs). AGEs are recognised by receptors for AGEs (RAGEs) expressed upon macrophages and their activation results in M1 polarisation and increased transcription of TNF and IL-1β via NF-κB [37]. A similar effect occurs due to FFAs. Prolonged periods of overnutrition initially lead to healthy adipose expansion, but when this capacity becomes exceeded, adipocytes are no longer able to safely store lipids and protect other tissue from their deleterious effects as excess lipids remain acellular in the form of FFAs. FFAs bind to TLR4, resulting in M1 macrophage activation and pro-inflammatory cytokine production [38].

### The influence of adipokines on macrophage polarisation

Leptin, the first adipokine discovered, plays a critical role in controlling food intake through central mechanisms. In addition, it is now considered to have an inflammatory role. Leptin activates the JAK2-STAT3 and PI3K-AKT-mTOR pathways in macrophages to promote a pro-inflammatory phenotype with the secretion of TNFα and IL-1β [39]. Leptin concentrations in the synovial fluid of OA patients correlate with BMI [40]. In addition to adipose tissue, leptin is produced locally within the joint by the cartilage, IFP, and synoviocytes [40], and leptin levels are significantly higher in the synovial fluid than in the serum of OA patients [41]. Expression in cartilage is upregulated in OA [40] and correlates with BMI of the patient [41], suggesting an important role for locally increased leptin production by joint tissues. In support of the clinical relevance of leptin in OA development, leptin serum levels 10 years prior to MRI assessment were associated with cartilage defects, bone marrow lesions, osteophytes, meniscal abnormalities, synovitis, and joint effusion in a population of middle-aged women [42]. These findings provide a strong indication for a role of leptin in the pathophysiology of OA.

Adiponectin, another adipokine produced by adipose tissue, has also been shown to influence macrophage polarisation state. Macrophages activated by M2 stimulants, IL-4 and IL-13, were shown to have increased AMPK activity and fatty acid oxidation when exposed to adiponectin. This resulted in increased levels of IL-10—a hallmark of M2 macrophage effector function. However, adiponectin also appeared to promote TNF, IL-6, and IL-12 production when macrophages were exposed to M1 polarising conditions [43]. In contrast, in a series of in vitro experiments, adiponectin was shown to promote re-polarisation of M1 macrophages towards an M2 phenotype, indicating a possible role in the resolution of inflammation [44]. Accordingly, a longitudinal study reported that OA progressed more slowly in patients with higher levels of adiponectin within their synovial fluid. Interestingly, adiponectin levels were inversely proportional to patients BMI [45]. This inverse relationship between adiponectin levels and BMI may be explained by adiponectin production being sensitive to both oxidative stress and fibrosis occurring in unhealthy adipose tissue expansion associated with obesity [46]. Thus, obesity and MetS downregulate one of the adipokines that may confer protection against OA via its effects on the innate immune system. However, another study showed that plasma adiponectin levels and adiponectin production by OA cartilage positively correlated with OA severity in a cohort of 35 patients undergoing total knee replacement surgery [47]. The role of adiponectin in OA pathophysiology thus remains to be clarified.

## The effect of MetS on chondrocytes

The metabolic perturbations associated with MetS, in addition to influencing macrophage polarisation and activity as outlined above, can contribute to OA pathogenesis by directly affecting chondrocytes. Both decreased AMPK and hyperactivation of mTORC1 resulting from MetS can negatively affect chondrocytes. A recent study in cartilage-specific AMPK knockout mice demonstrated increased degradation of cartilage in both age-related OA and post-traumatic OA due, at least in part, to the loss of protection from the catabolic effects of IL-1β activating NF-ϰB and resulting in the production of MMPs [48]. This has been corroborated by the selective AMPK activator, A769669, shown to significantly reduce cartilage breakdown in human chondrocytes exposed to IL-1β and TNF [49]. mTORC1 hyperactivation has been implicated in the development of OA through its suppression of autophagy. Autophagy, as a mechanism for recycling damaged cellular organelles, is vital for cell survival. Rapamycin blockade of mTORC1 activity has been shown to significantly increase autophagy within articular chondrocytes and reduce OA severity, accompanied by reductions in both synovitis and ADAMTS-5 expression in the articular cartilage [50]. Elevated levels of FFAs may also directly affect chondrocytes within the OA joint. When human chondrocytes are cultured in the presence of saturated FFAs, it results in the increased expression of the inflammatory cytokines IL-6 and IL-8. Concurrently, superoxide radical, reactive nitrogen species, and hydrogen peroxide were all upregulated within the human chondrocytes [51]. Furthermore, leptin has been shown to affect chondrocytes via its ability to stimulate chondrocytes to produce numerous catabolic and inflammatory factors. Gene expression analysis of cartilage from rats with leptin-induced OA and healthy controls revealed increased expression of genes encoding for MMPs, inflammatory cytokines, and apoptotic factors in the leptin-induced OA group [52]. Similarly, human chondrocytes stimulated with leptin upregulate MMP1, MMP3, and MMP-13 [53], and increase nitric oxide synthase type II when leptin is combined with IL-1β [54]. Finally, leptin has been reported to induce cell senescence in chondrocyte progenitors by activating the p53/p21 pathway and inhibiting Sirt1 (responsible for degrading p53), resulting in impaired ability to migrate and differentiate into chondrocytes [55]. Cell senescence is increased in OA cartilage, and senescence is emerging as an important player in OA pathogenesis. It occurs as a result of cell cycle arrest in response to cellular stressors, leading to cellular hypertrophy and resistance to cell death signals. Importantly, cell senescence contributes to chronic inflammation through promoting the senescence-associated secretory phenotype (SASP). Chondrocytes exhibiting SASP produce IL-1, IL-6, CCL2, and MMPs amongst other factors, leading not only to cartilage breakdown and synovitis but, in a paracrine manner, inducing further chondrocyte senescence [56]. The importance of these processes was demonstrated when senescent cell clearance, either through genetic ablation or treatment with the senolytic agent UBX0101, attenuated the development of OA in mice following ACL transection or with age [57]. Beneficial effects of UBX0101 treatment were also observed in human OA chondrocytes in vitro [57], and this agent is currently in a phase I clinical trial for knee OA (www.clinicaltrials.gov). Taken together, these data highlight the role of MetS on OA not only via the activation and polarisation of macrophages but also via direct detrimental effects on chondrocytes.

## Implications for OA treatment

As the body of evidence has built up implicating MetS in shaping our inflammatory response in the context of OA, an important question is whether weight loss and an associated reversal of MetS could lead to the halting of OA disease progression. Numerous studies have been conducted assessing the impact of weight loss on metabolic dysfunction with implications for macrophage activation and systemic inflammation. Diet-induced weight loss over a 3-month period significantly reduced circulating saturated FFA levels [58]. Weight loss has also been shown to significantly reduce circulating AGEs, demonstrated by reductions in HbA1c (glycated haemoglobin) [59]. As these are known to activate TLR4 and RAGEs, respectively, weight loss may decrease the activation and polarisation of M1 macrophages and hence reduce inflammation. In support of this, OA patients experiencing weight loss following bariatric surgery had significantly reduced serum leptin, IL-6, and high-sensitivity C-reactive protein levels, and increased serum adiponectin levels [60]. This was associated with decreased pain and improved function scores, as well as increased levels of N-terminal propeptide of type IIA collagen, indicative of cartilage production, and decreased levels of cartilage oligomeric matrix protein, indicative of cartilage degradation [60]. Weight loss can thus decrease systemic inflammation and alleviate symptoms of knee OA.

In addition to weight loss, there has been interest in drugs such as metformin, used in the treatment of diabetes for many years. Metformin primarily acts to reduce hepatic gluconeogenesis whilst also increasing glucose utilisation by the intestine, thus reduce AGE formation implicated in the activation and polarisation of M1 macrophages [61]. Furthermore, its molecular mechanism of action involves activating AMPK [61], which may have further protective roles in the context of OA, as discussed above. Metformin has recently been used in a prospective cohort study and was shown to reduce cartilage loss in OA patients over a 4-year period [62]. Whilst it did fail to reach significance for the reduction in total knee replacement after 6 years, this may be due to the relatively small number of participants in the study who were currently taking metformin and thus remains a promising avenue for future research to determine the influence metformin may have on OA progression. It must also be acknowledged, however, that there are drawbacks to metformin use. Significant proportions of patients started upon the drug are unable to tolerate the side effects that often accompany its initiation, most notably gastrointestinal upset [63].

Whilst old drugs such as metformin could potentially be repurposed for treating OA, there is a need for further strategies to combat OA. One such strategy is utilising dietary ω-3 PUFA derivatives to influence macrophage polarisation and OA disease progression. Mice fed different ratios of ω-6 polyunsaturated fatty acids (PUFAs) compared to ω-3 PUFAs to induce obesity showed significant differences in the severity of OA, synovitis, and wound healing. Those with greater levels of ω-6 PUFAs had significantly worse outcomes, as well as increased leptin and decreased adiponectin levels [64]. However, this is controversial with others demonstrating mice fed with a ω-6-rich diet over a 24-week period to have no increased risk of synovitis when compared to those fed with ω-3 PUFAs [65]. Despite these differing results, PUFA derivatives have been trialled therapeutically. The ω-3 PUFA derivative resolvin D1 (RvD1) has been reported to re-polarise macrophages to an M2 phenotype with decreased production of IL-8, IL-1β, and CCL2 [66]. Mice receiving a HFD and treated intra-articularly with RvD1 showed decreased susceptibility to post-traumatic OA compared to mice injected with vehicle [67]. This provides insight that RvD1-like molecules could mediate re-polarisation of macrophages and reduction in inflammation. Results from a 2016 clinical trial showed oral ω-3 to be beneficial in reducing patient pain scores in OA but failed to demonstrate any benefit in reducing cartilage loss [68]. Further investigation is therefore warranted to determine whether, with a potential intra-articular route of administration, OA progression could be slowed, thus paving the way to a potential DMOAD.

## Conclusions

Our understanding of the pathogenesis of OA has come a long way from the long-standing paradigm of a disease caused by ‘wear and tear’. A plethora of new evidence has emerged highlighting the importance of chronic, low-grade inflammation in the pathogenesis of this debilitating condition. Macrophages, as crucial mediators of the innate and adaptive immune response, have been extensively studied, and it is now clear that an imbalance in macrophage phenotype is contributing to this condition. Given the association of OA with obesity in an increasingly overweight population, the impact of metabolic factors on the development of joint disease has become an area of intense investigation. As such, dyslipidaemia, hyperglycaemia, and aberrant adipokine secretion have emerged as important metabolic regulators capable of influencing the chronic inflammation seen in OA. These discoveries reaffirm the role weight loss plays in the management of OA, how weight loss could per se result in resolving inflammation, metformin can alter metabolic regulators, and how dietary fatty acids might be promising targets for DMOADs. Whilst new therapies will require substantial further work to reach fruition, the studies reviewed here offer significant encouragement that novel treatments will emerge for this prevalent and debilitating condition.

## Data Availability

Not applicable

## Abbreviations

ADAMTS: A Disintegrin and Metalloproteinase with Thrombospondin Motif
AGE: Advanced glycation end-product
AMPK: 5′ Adenosine monophosphate-activated protein kinase
BMI: Body mass index
BMPs: Bone morphogenetic proteins
CCL: Chemokine ligands
CD: Cluster of differentiation
DAMP: Damage-associated molecular patterns
FFA: Free fatty acid
G6PD: Glucose-6-phosphate-dehydrogenase
HFD: High-fat diet
HIF: Hypoxia-inducible factor
IFP: Infrapatellar fat pad
IL: Interleukin
IRAK-M: IL-1 receptor-associated kinase M
LOXL3: Lysyl oxidase-3
MetS: Metabolic syndrome
MMP: Matrix metalloproteinase
MRI: Magnetic resonance imaging
mTORC1: Mammalian target of rapamycin complex 1
NO: Nitric oxide
OA: Osteoarthritis
PPP: Pentose phosphate pathway
PRR: Pattern recognition receptor
PUFA: Polyunsaturated fatty acid
RAGE: Receptor for AGE
ROS: Reactive oxygen species
RvD1: Resolvin D1
SASP: Senescence-associated secretory phenotype
SCECT-CT: Single-photon emission computed tomography-computed tomography
TGF: Transforming growth factor
TLR: Toll-like receptor
TNF: Tumour necrosis factor
VEGF: Vascular endothelial growth factor
